# Substance use care innovations during COVID-19: barriers and facilitators to the provision of safer supply at a toronto COVID-19 isolation and recovery site

**DOI:** 10.1186/s12954-024-00935-w

**Published:** 2024-01-20

**Authors:** Gillian Kolla, Chowdhury Nishwara Tarannum, Kaitlin Fajber, Fiqir Worku, Kris Norris, Cathy Long, Raphaela Fagundes, Anne Rucchetto, Eileen Hannan, Richard Kikot, Michelle Klaiman, Michelle Firestone, Ahmed Bayoumi, Gab Laurence, Kate Hayman

**Affiliations:** 1 Canadian Institute for Substance Use Research, University of Victoria, Victoria, BC Canada; 2 MAP Centre for Urban Health Solutions, St. Michael’s Hospital, Unity Health, Toronto, ON Canada; 3 Dalla Lana School of Public Health, University of Toronto, Toronto, ON Canada; 4 The Neighbourhood Group, Toronto, ON Canada; 5 Parkdale Queen West Community Health Centre, Toronto, ON Canada; 6 Department of Emergency Medicine, St. Michael’s Hospital, Unity Health, Toronto, ON Canada; 7 Faculty of Medicine and Institute of Health Policy, Management and Evaluation, University of Toronto, Toronto, ON Canada; 8 Division of General Internal Medicine, St. Michael’s Hospital, Unity Health Toronto, Toronto, ON Canada; 9 University Health Network, Toronto, ON Canada; 10 Faculty of Medicine, University of Toronto, Toronto, ON Canada; 11 Memorial University, St. John’s, NL Canada

**Keywords:** Safe supply, Safer supply, COVID-19, Isolation site, Substance use, Overdose, Shelter, Opioid use disorder

## Abstract

**Background:**

Early in the COVID-19 pandemic, there was an urgent need to establish isolation spaces for people experiencing homelessness who were exposed to or had COVID-19. In response, community agencies and the City of Toronto opened COVID-19 isolation and recovery sites (CIRS) in March 2020. We sought to examine the provision of comprehensive substance use services offered to clients on-site to facilitate isolation, particularly the uptake of safer supply prescribing (prescription of pharmaceutical opioids and/or stimulants) as part of a spectrum of comprehensive harm reduction and addiction treatment interventions.

**Methods:**

We conducted in-depth, semi-structured interviews with 25 clients and 25 staff (including peer, harm reduction, nursing and medical team members) from the CIRS in April–July 2021. Iterative and thematic analytic methods were used to identify key themes that emerged in the interview discussions.

**Results:**

At the time of implementation of the CIRS, the provision of a safer supply of opioids and stimulants was a novel and somewhat controversial practice. Prescribed safer supply was integrated to address the high risk of overdose among clients needing to isolate due to COVID-19. The impact of responding to on-site overdoses and presence of harm reduction and peer teams helped clinical staff overcome hesitation to prescribing safer supply. Site-specific clinical guidance and substance use specialist consults were crucial tools in building capacity to provide safer supply. Staff members had varied perspectives on what constitutes ‘evidence-based’ practice in a rapidly changing, crisis situation.

**Conclusion:**

The urgency involved in intervening during a crisis enabled the adoption of prescribed safer supply, meeting the needs of people who use substances and assisting them to complete isolation periods, while also expanding what constitutes acceptable goals in the care of people who use drugs to include harm reduction approaches.

## Introduction

Similar to many jurisdictions responding to the urgent threat posed by the spread of the novel SARS coronavirus-2, on March 17, 2020, the province of Ontario declared a state of emergency due to COVID-19 and initiated public health measures such as lockdowns, service closures, physical distancing and stay at home orders to mitigate the spread of COVID-19. As a result, numerous health and social services were temporarily closed and made inaccessible to many who urgently needed them, including people who use drugs and people experiencing homelessness. The COVID-19 pandemic occurred against the backdrop of a deadly crisis due to a toxic drug supply in Canada: almost 35,000 drug-related overdose deaths occurred between January 1st, 2016 and September 2022, of which 81% involved fentanyl [1].

The COVID-19 pandemic exacerbated existing health disparities for people who use drugs, particularly among those experiencing homelessness [2, 3]. The overall number of opioid-toxicity related overdose deaths in Ontario increased by 79% from February 2020 (the month before COVID pandemic measures were introduced in the province) to December 2020, while deaths from opioid-toxicity among people experiencing homelessness increased by 129% [4]. Importantly, the concurrent public health emergencies of the COVID-19 pandemic and the overdose crisis occurred within the context of a severe lack of affordable housing in many cities in Canada. Concerns regarding the quick spread of COVID-19 in congregate settings–including the homeless shelter system–prompted the establishment of spaces for individuals who were experiencing homelessness, unsheltered, and/or living in shelters or encampments to isolate when exposed to or infected by COVID-19. As such, in March 2020, a collaboration between community agencies and the City of Toronto led to the development of COVID-19 Isolation and Recovery Sites (CIRS), which opened in a hotel in Etobicoke on April 9th, 2020 [5].

Hotel-based isolation sites in the USA were associated with completion of mandated COVID-19 isolation and safely supporting people who are experiencing homelessness, while also decreasing the number of emergency department visits, hospitalizations, inpatient hospital days, and psychiatric visits [6, 7]. In Canada, the prescription of pharmaceutical opioid and stimulant medications as safer supply was provided alongside traditional addiction treatment and managed alcohol programs at temporary isolation sites, and was associated with both successful adherence to mandatory isolation and prevention of overdoses, with very few instances of over-intoxication or diversion documented [8, 9]. Prescription of a regulated source of opioids and/or stimulants is a modality of safer supply, which has been defined as providing access to a “legal and regulated supply of drugs with mind/body altering properties that traditionally have been accessible only through the illicit drug market” [10]. Safer supply prescribing began in Ontario on a small-scale prior to the COVID-19 pandemic, with 447 unique individuals receiving prescriptions for take-home doses of immediate release hydromorphone as safer supply from 2016 to 2020 [11]. The unique circumstances presented by a co-occurring pandemic and overdose crisis led to an acceleration in the scale-up of these programs during the early months of the COVID-19 pandemic in Canada [12, 13].

Across Canada, research and evaluation of safer supply programs have demonstrated numerous positive impacts for individuals, as well as ongoing challenges at the program and system levels. At the population level, an analysis of health administrative data found rapid and statistically significant reductions in emergency department visits, hospital admissions, and health care costs (excluding primary care and medication costs) among clients enrolled in a safe supply program in London, Ontario, while no change in these outcomes was observed in a matched comparison group of London residents with opioid use disorder who did not receive safer supply [14]. Safer supply clients across Canada have reported outcomes of improved self-reported health and stability; improved relationships with friends and family; reduced rates of overdose; reduced reliance on the unregulated drug supply; reduced use of injection to administer drugs and reduced harms associated with injection drug use; and reduced involvement in criminalized activities, street hustles, and sex work [15–22]. Service providers workings in safer supply programs have echoed seeing positive impacts on client health and stability, and have also highlighted challenges with meeting client needs due to temporary funding sources, limited program capacity, restricted medication options available under provincial drug formularies (specifically no option that can be smoked), and fears of repercussions from other clinical staff and/or regulatory colleges for providing safer supply [23–25]. Health planners reflect that enduring stigma and criminalization of drugs and people that use drugs led to the scale-up of medicalized models for safer supply, which has limited availability and accessibility of safer supply programs that meet a variety of community needs [26]; they also emphasize the need to tailor services to the individual and local contexts, emphasizing that there is no “one size fits all” approach [27]. There is a paucity of research specific to understanding provider perspectives on safer supply and how those perspectives shape willingness and capacity to prescribe safer supply as an alternative to the unregulated supply. At the time the CIRS opened in early April 2020, prescribing opioids for unsupervised use as ‘safer supply’ was a contentious and much-debated subject among clinicians providing care to people who use drugs. Notably, six Canadian Society of Addiction Medicine board members had published a letter to the editor raising “grave concerns” about the model prior to the pandemic [28], highlighting the strong debate surrounding this practice within the Canadian addiction medicine community during this period. Despite the ongoing debate, the public health challenges presented by the COVID-19 pandemic prompted two Canadian provinces to release interim guidance on “Risk Mitigation Prescribing” in 2020 [29, 30]. This variation of safer supply provided take-home doses of medications to facilitate isolation of people who use drugs in the early pandemic period, and contributed to rapid uptake of safer supply prescribing in early 2020 [12].

Given the growing evidence demonstrating positive outcomes for safe supply clients, ongoing challenges in initiating these programs and the continuing debate on the role of safer supply prescribing, this study is designed to examine the barriers and facilitators to rapidly establishing safe supply programs in an emergency context during the onset of COVID-19 emergency measures in March 2020. At the time, safer supply was a relatively novel intervention and the evidence base that now exists was still emerging. Using data from qualitative interviews with CIRS clients and staff, we aim to identify the resources and policy changes that are necessary to accelerate the establishment and expansion of safe supply programs amid multiple deadly crises.

## Methods

### Program description: Substance Use Services at the Toronto COVID-19 Isolation and Recovery Site

Due to the strong need to facilitate isolation in the early days of a global pandemic and the recognition of the ongoing overdose crisis, the Substance Use Services at the CIRS included a comprehensive array of harm reduction and treatment interventions to assist people to complete the mandated isolation period (see Table 1). Notably, this included providing a prescribed safer supply of opioids and/or stimulants as an alternative to unregulated use of drugs that people would buy for themselves, either alone or alongside traditional opioid agonist treatments, to prevent withdrawal, overdose and departure from the CIRS before completing the mandated isolation period. The Substance Use Services aimed to provide comprehensive and personalized clinical and harm reduction supports for people who use substances in a space that also facilitated COVID-19 related care goals. Substance use services were delivered by an interdisciplinary team that included peer workers, harm reduction workers, nurses, primary care providers (nurse practitioners, family and emergency medicine physicians), and specialist substance use physicians [5].

**Table 1:** Substance use services provided at the CIRS

Harm reduction education and distribution of harm reduction equipment (including sterile injection equipment, and safer smoking and inhalation equipment)
Provision of cigarettes and outdoor space for physically-distanced smoking
A managed alcohol program
Prescription opioids and/or stimulants as treatment or as an alternative to unregulated drugs that people would buy themselves (opioid agonist treatments (OAT), safer opioid supply (SOS), stimulant medications)
Prescription of medications to treat withdrawal from drugs or alcohol
Services to prevent and respond to overdoses:
An on-site overdose prevention site (a room where people can go to use substances—primarily by injection—under the supervision of trained staff)
In-room witnessing when using substances by staff when clients requested it
Telephone or in-person check-ins when using substances when clients requested it
Naloxone training and distribution to staff and clients

### Data collection

Our research team recruited 25 clients and 25 staff from the CIRS to complete qualitative interviews as part of a multi-stakeholder evaluation of the substance use services that operated at the CIRS from April 9th, 2020 to June 30th, 2021 at a hotel in Etobicoke, Canada. Interview guides were developed with input from by the community-based research team, which included front-line staff members in multiple roles from the CIRS and members of the leadership team (including staff who had been/were members of the peer team, the harm reduction team, the nursing team and the physician team). Additionally, community stakeholders and members of the research team had participated in the development of a logic model that helped determine major areas of inquiry for the interview guide [5]. The interview guide for clients explored: experience prior to coming to the CIRS; drug use and experience of overdose at the CIRS; access to and experience of substance use services at the CIRS (including experience with service providers), and; discharge from the CIRS. Interview guides for staff members mirrored some elements of the client interview guide, but also consisted of questions on communication and dynamics of collaboration between the interdisciplinary staff teams working at the CIRS. Major topic areas explored in staff interview guides included: staff roles, perspectives of and previous experience with harm reduction; experience providing substance use services at the CIRS (including overdose response); discharge planning; and site operations, including communication, decision making and collaboration between interdisciplinary teams onsite.

Clients were recruited in April and May 2021 and were asked to complete in-depth, semi-structured qualitative interviews. During the months of April and May 2021, Toronto was in the midst of the 3rd wave of COVID-19, with lockdowns in effect and severe outbreaks of COVID-19 within homeless shelter settings across the city. Due to this, the CIRS was operating at or near capacity (approximately 150 clients) during participant recruitment. More men were in residence during this period than people identifying as women, non-binary, or another gender; our team attempted to oversample women and people with non-binary genders when possible to ensure the experiences of clients of various genders were represented. All participants were current clients of the CIRS at the time of their interview, although the length of time at the site varied. Clients receiving substance use services were identified by on-site partners, and clients who expressed interest in participating in the study were referred to study staff. Peer researchers who either currently or previously had worked on one of the teams at the CIRS contacted potential participants to confirm interest and eligibility, obtain informed consent, and complete the interviews. A second research team member was available during the interviews so that peer researchers did not consent or interview clients to whom they were also providing direct peer support. Peer researchers supported participants with setting up a tablet in their rooms, and then moved to a separate room to conduct the interview over Zoom.

Staff interviews took place in June and July 2021. While staff did not have to be currently employed at the CIRS at the time of their interview, they had to have worked frequent shifts for at least one month to ensure they had the depth of experience necessary to comment on the workings of the Substance Use Service. A purposive sampling strategy was used to recruit 5 site staff from each of the 5 teams involved in the provision of substance use services: peer workers, harm reduction workers, nurses, primary care providers (nurse practitioners, family and emergency medicine physicians), and specialist substance use physicians. Community partners from 3 of the 4 main organizations involved in delivering services at the CIRS were part of the research team; these partnerships were leveraged to identify potential candidates for staff interviews. Community partners were asked to contact potential participants and obtain their consent to pass on their contact information to research staff, who then reached out by email to recruit participants.

Study staff provided all study participants with the study’s consent form ahead of the interview and offered an opportunity to ask any questions of the study team prior to the interview. All clients and staff were interviewed over Zoom. With the consent of participants, all interviews were audio-recorded and transcribed, with any identifying details removed during transcription. Interviews with clients ranged in length from 23 to 66 min, lasting an average of 35 min. Interviews with staff ranged in length from 43 to 94 min, lasting an average of 70 min. Participants were offered a $40 honorarium for their participation in the qualitative interview. Unity Health Toronto Research Ethics Board approved this study.

### Analysis

The full research team–which includes members of the on-site frontline and leadership teams at the CIRS—provided input into the main thematic areas to focus on during analysis. An analysis team composed of research coordinators, research assistants and one of the project leads (who was not an on-site staff member or involved in directly providing care to clients or supervision to staff) met regularly and were responsible for developing the analytic plan and conducting analyses. Analysis team members coded and analyzed all transcripts using Dedoose (www.dedoose.com). To maintain confidentiality of the participant responses, community partners on the research team did not have access to audio-recordings or transcripts. Iterative and thematic analytic methods were used to identify key themes that emerged in the interview discussions along with themes that were identified from feedback from the full research team on main areas of analytic interest. Once initial themes were identified, they were compared between the different groups of participants to identify consistent themes. Analysis team members met regularly to talk about emerging themes and identify the main areas for analytic attention. They also regularly presented preliminary results from the coding and analysis to the full research team for comment, feedback, and refinement.

## Results

The demographic characteristics of clients and staff members who participated in interviews are presented in Tables 2 and 3. Our results focus on: the major reasons identified by staff members for prescribing safer supply to clients; the major sources of hesitation they experienced while integrating this novel practice; the methods used to build staff capacity for providing safer supply; and the ways that expertise of clients who use drugs was centered. Finally, we explore the ways staff members–particularly clinical staff–reflected on what constitutes ‘evidence-based’ practice, and how this practice may shift in a rapidly changing, crisis situation.

**Table 2:** Participant demographics—CIRS Clients

	Clients (*n* = 25)
Gender	
Women	7
Men	16
Transgender, gender-fluid, gender non-conforming, or non-binary	2
Racial/Ethnic Identity	
Black, Indigenous or other Racialized Identity	13
White	12

**Table 3:** Participant demographics—CIRS Staff Members

	Staff members (*n* = 25)
Gender	
Women	16
Men	7
Transgender, gender-fluid, gender non-conforming, or non-binary	2
Racial/Ethnic Identity	
Black, Indigenous or other Racialized Identity	10
White	15
Staff team	
Peer worker	5
Harm reduction worker	5
Nurse	5
Primary care provider (Nurse practitioner, general or emergency medicine physician)	5
Substance use physician	5

### Reasons for integrating safer supply in the isolation sites

The CIRS model integrated opioid, alcohol and stimulant replacement options into temporary shelter spaces to facilitate the completion of COVID-related isolation periods for people experiencing homelessness. The importance of the provision of prompt and comprehensive substance use services was paramount to facilitating COVID-19 related isolation, reflected in one client's statement: “If I didn't get my medication, like I would have been a hundred percent gone”. (*CIRS Client receiving safer opioid supply*).

The unique circumstances of the COVID-19 pandemic created an urgent need to provide harm reduction and substance use supports directly within the CIRS and allow clients autonomy to use substances on-site. Without access to these services, they would have been prompted to leave the site to seek out or use substances elsewhere despite wanting to maintain COVID-related isolation to prevent community transmission:

“If I had withdrawal issues with alcohol or the opioids and they just didn't want to do anything about it, I would have checked myself out. I wouldn't have felt good about it because I'm trying to be responsible. I don't want to spread the COVID. I would have done my best to be careful. But no, I would have to leave to get myself my alcohol and get myself my drugs.” (*CIRS Client receiving safer opioid supply and the managed alcohol program*)

Some clients stated that they were not interested in a conversation regarding their substance use but found value in the on-site supports and services. A clinical staff member recalled how this would come up in the intake process, when they would interview clients to assess their need for services and some clients would not be interested in long-term supports around their substance use, and would voice a need for short-term supports related to their COVID isolation only: “…it's something that is just while they're isolating, they just need some support’’ (*Staff–primary care provider*). Traditional concepts of patient engagement in addiction medicine were challenged in this space, since patients were at the site related to their COVID-diagnosis, and not overtly seeking addictions or substance use treatment:

“Why is this so different than my addiction practice? It's like no one's coming to you for help. They're coming to you for withdrawal. And so there's not this obsession with buy-in and everything else. Some people are not interested [*in addictions treatment*] and that dynamic is very different. So I would say that piece is quite new to me.” (*Staff–primary care provider*)

Importantly, this represented a major shift in the context and care goals for most of the prescribers, who might otherwise engage with clients only when they would self-identify a strong desire to change their substance use or engage in addiction treatment. One prescriber describes how using safer supply as a form of substitution therapy (in this case, with stimulants) felt more appropriate in this clinical setting:

“It's a big departure from the framework of addiction medicine, whereby it's typically as a treatment. And so many people like to cite the fact that there is not great evidence to support the use of stimulants in the doses that have been prescribed in trials for treatment. Now, that is the lens that one is going for in terms of complete abstinence, but then shifting that focus to people need to isolate so how do we best support them? I think it kind of opened up that freedom to just feel like we could try prescribing this.” *(Staff–substance use physician)*

For many of the providers interviewed, this dynamic where clients at the CIRS were not seeking out treatment for addictions or substance use but were instead being forced to isolate–often on short notice and without access to their drug(s) of choice–in the context of an evolving public health emergency resulted in a willingness to prescribe safer supply.

### Overcoming prescriber hesitancy

Some of the initial hesitations with prescribed safer supply stemmed from a lack of clinical experience and evidence around safer supply at that time, and the sense that it was a controversial practice:

“At the beginning, it kind of felt a lot of us - including me - were very uncomfortable with (*safer*) opiate prescribing because, number one: we'd never done it before. Number two, there's still a lot of controversy around it.” *(Staff–substance use physician)*

Prescribers and nurses highlighted the tension they felt when engaging in a practice that they believed may be controversial in the eyes of their Regulatory College: “But the hesitancy came down to this is my license. It wasn't the patients. Are they going to be safe? It was my license”* (Staff–nursing team).* They also describe the difficulties with initiating prescribing of safer supply when many leaders in the field of addiction medicine were actively opposing it, particularly when those people had been their mentors:

“And the other conflict is the fact that this is an unsupported practice. So, you feel like as a physician and as a young physician who has always looked up to this person as a mentor who was instrumental my training. Actually, it's hard to go against that sometimes.” (Staff–substance use physician)

The harm reduction approach was novel to many of the clinical team members, as was tolerance for drug use on site. When speaking about the use of drugs like unregulated fentanyl at the site by clients, one nurse describes how:

“In hospitals and most clinical care settings, essentially the culture is, ‘Don’t let something happen on your watch’. Your job as a nurse is to assess someone and recognize the subtle cues before something serious happens, so that you’re actually preventing an incident from occurring in the first place. And so coming from that mindset, and then having someone engaging in a risk behavior that could lead to serious harm or death…It's very challenging for nursing.” *(Staff–nursing team)*

Despite initial hesitations around prescribing safer supply, physicians and nurse practitioners at the CIRS were motivated to expand their clinical practice to include it. This may have been motivated, in part, by concerns over the risk of on-site overdose, which was a dominant theme in interviews. Staff at the CIRS discussed directly witnessing harms related to the toxic drug supply, including having to respond to overdoses at the site. Peer workers were often the first responders to overdose on site, describing the frequency and urgency of overdose response: “We started carrying Naloxone with us 24–7 after we had, I think, 7 close calls throughout the day over a shift?” (*Staff–peer team*). Due to the high frequency of overdose response incidents, staff became very adept at responding to overdose and resuscitating clients when necessary: “So we do a really, really good job from that regard in terms of overdoses and responses. And you can't imagine how quickly front-line workers run from each department to that spot to provide support.” (*Staff–peer team*).

The unique environment of the CIRS, with clients isolating in hotel rooms both to allow for dignity while they were recovering and to prevent the transmission of COVID-19, presented a challenge to overdose prevention and response, which often relies on observing people using drugs to allow for rapid response in the event of an overdose. Many clients brought drugs they had purchased prior to entering the CIRS with them and, despite the presence of an on-site OPS and in-room spotting, did not disclose their drug use to staff. Staff members highlighted the challenges of supporting clients to use more safely while also supporting client autonomy, and how this relates to the emotional impact of responding to overdose:

“And of course, you don't want to find when your clients dead, like nobody does. But I think that, it was a real moral struggle around how do we how do we respond from a client centered point of view, respecting their autonomy and finding some kind of safety balance for folks that we know are using high and frequent amounts of fentanyl on site, because that's a real risk.” *(Staff–harm reduction team)*

Faced with having to personally respond to so many overdoses, the risks of not prescribing safer supply as an alternative to fentanyl from unregulated street markets felt immediate to many of the team members supporting clients at the site. As one prescriber stated: “I think one of the things in medicine that is quite pervasive is the idea of doing no harm. But we don't often talk about the harm in the things we don't do. And when the risks of not intervening in the midst of the fentanyl crisis are incredibly high.”* (Staff–substance use physician).*

Prescribers were also motivated by the opportunity to engage with clients who might not otherwise interact with the healthcare system. This dilemma was discussed by a physician who stated that: “I think each of us had a sense of like, this is our only shot.”* (Staff–substance use physician)*. Although the reason for their stay was COVID-related isolation, healthcare providers recognized the potential of the CIRS stay as a moment of engagement with the healthcare system for people who were often disconnected from healthcare services:

“Ideally, the medical system should be improving your wellness. And I think that there was a lot of feeling that just by improving the dignity and human compassion, that you were improving their lives and their engagement in resources.” *(Staff–substance use physician)*

### Building confidence

Prescribers also required concrete tools to develop their confidence and knowledge about the specifics of prescribing safer supply, as well as the broader array of medications used in traditional addiction treatment. Importantly, these tools needed to be adapted to the unique operational differences between their current setting–a hotel re-purposed as an isolation site for people experiencing homelessness—and the outpatient addiction medicine, emergency department, and inpatient settings in which they typically practiced. As one prescriber described:

“Oftentimes, I wasn't the one getting the full history. And so…there's always a bit more of a discomfort. We were doing no urine drug screens, which is, again, a bit of a departure. And mostly my concern around that was someone who maybe their substance use wasn't entirely clear. And usually, I use a urine drug screen to be like, ‘Oh, yes, it's positive for fentanyl. I know your tolerance is OK’. So that was it was just a little uncomfortable in the beginning and just different.” *(Staff–substance use physician)*

There were two major factors that prescribers identified as helping to support them to build competence for prescribing at the site: the development of a written guidance document for prescribing specific to the CIRS, and the availability of an on-call substance use specialist service for consultation as needed. Written guidance was described as helpful in establishing within-group norms for prescribing at the site:

“We actually had a document, it's like, okay, this is developed by physicians and not just physicians, but there's a whole team of various people who developed this document, including people with lived experience. So it was like, okay, we have this document to support us in our practice, developing a standard of care so that we're not kind of randomly prescribing safer opiates, applying and getting ostracized by the [*addiction*] medicine community in this province. So as we have a document, I think a lot of us were just a lot more comfortable with it because we had something down on paper.” *(Staff–substance use physician)*

This document was developed with support from community-based safer supply prescribers in the city, who also provided support through meetings and workshops for team members. Despite some continuing hesitation and concern about the lack of concrete data on this approach, team members attended these trainings and were open to learning.

“And then to be honest, it was just listening to people. So it was coming to these meetings with the nurse practitioners and the people from [*name of local supervised injection site*] and listening to other people who are who had already been doing this. We had a meeting with [*name of safer supply prescriber*] right at the beginning. And we were listening to the people who really were on the ground. And so hearing their opinions about why this is so important really was comforting to me and sort of pushed me to reconsider my own position. Because, to be honest, I don't know if I'm still convinced that it reduces fentanyl use. I'm not sure. And we don't really have any good data yet to tell us that it's helpful. But it seems to be–logically - it seems to be the right thing to do, right? It seems we need to provide people with a safer opiate supply so they're not using fentanyl. Whether that's actually happened, I don't know. But that's the intention.” *(Staff–substance use physician)*

In addition to the development of a guidance document and consultations, a team of substance use specialists were available to support the on-site primary care providers at the CIRS. Access to the substance use specialist physicians was a valuable tool to support the upskilling of on-site prescribers, who regularly consulted with them to inquire if they were on the right track. One substance use specialist described their role at the CIRS as including:

“Providing phone calls, support to the physicians and the nurse practitioners working in that setting. So that involves them calling me if they have a question specifically about substances and then occasionally that involves me contacting the clients directly and doing care to them and then prescribing medications to them.” *(Staff–substance use physician)*

Access to substance use specialists, which provided the ability to consult with experienced colleagues to develop a collaborative care plan, was highlighted by the on-site prescribers as a major facilitator to the development of expertise in this area: “And you do need…like you need a thought leader. You need backup in your decision making. I called [*name of substance use specialist on call*] at 11:00 last night…because, I just need someone to back me up.” (*Staff–primary care provider*).

The support of the specialist substance use team also helped on-site primary care providers to develop comfort with the high doses required to support people with heavy exposure to fentanyl from unregulated markets. Many staff members described this as a process of building comfort over time:

“I think it took some time for us to really get rocking and rolling with risk mitigation prescribing. I think it took us a long time and it definitely took a lot of prescribers a long time to get comfortable with the doses, and like just like how much we could tap in with the doses, so we had sort of a transition.” (*Staff–primary care provider*)

Members of the substance use specialist team also described how consultations shifted through time as the on-site prescribers gained experience and confidence in prescribing medications for substance use. They described a progression from wanting detailed advice around prescribing to moving toward quick check-ins for reassurance: “Now pretty much when they call me, I'm just like, ‘that's an amazing plan’. They still want a little bit of reassurance that what they're doing is great. Whereas before I would have more suggestions or I’d say, ‘OK, well, did you ask about this?”* (Staff–substance use physician).*

Beyond consultation with the team of substance use specialists who were on call to support on-site clinical staff, the interprofessional nature of the site also supported capacity building around harm reduction more generally. As one nurse described:

“I learned to really rely heavily on peers and harm reduction because I really see them as the expert. They sort of gave me the permission to step back. I would sort of rely on them to tell me where my boundaries should be with safety checks. If that makes sense, sort of saying like, ‘how do you feel we should be approaching this client in terms of harm reduction and safety?’ And how far do we take it from your perspective and then sort of just go based on their expertise? And also they have more of an ability to develop a different relationship with clients.” *(Staff–nursing team)*

The dynamic and multifactorial environment that was driving the change in practice that led to building confidence around prescribed safer supply and the integration of harm reduction more broadly was summarized by another of the prescribers:

“It definitely has a little bit of everything. A lot of interactions with clients and just building that rapport and relationships with them. But it also had a lot to do with the trainings that were provided on site, because it is an evolving topic and it's always ever changing. So there was a lot offered, a lot of resources that were offered. And even like the managers who've been in this sector for so many years, they're also willing to kind of share their experience and their views. And then, you know, it's just like having a conversation about it. And that's how I was able to learn so much about it.” *(Staff–harm reduction team)*

### Centering the expertise of clients

The heavy focus on harm reduction at the site–including the centrality of the peer and harm reduction team in services delivery–helped to facilitate the centering of the expertise and experience of clients and people who use drugs. Prescribers highlighted how their interactions and conversations with clients was crucial in the development of their practices at the CIRS.

“Just listening to people, people's own experiences really helped kind of sway me a little bit. I'm still going to be - I have to be fair. I have to be honest, like I'm still a little bit conflicted about it. I don't prescribe safer opiate supply out of my community practice because that specific organization is not on board with it. So that's more that might change. But I am starting to prescribe it.” *(Staff–substance use physician)*

Prescribers increasingly recognized that clients held significant expertise about their drug use, and were receptive to novel solutions that would facilitate on-site isolation and risk reduction. One physician described how the balance of expertise between patient and prescriber is often different when providing substance use care than in other areas of medicine:

“I think the way we are trained as physicians means that in most scenarios, we do have a lot of expertise to offer people. And if we think about a medical model, if you are going to have a heart attack in the hospital, chances are your cardiologist does know more about cardiac care and the heart than you do generally. That is very true. And I think if you are using fentanyl and you go to the hospital, you probably know more about using fentanyl than your provider. And even as an addiction provider - like you know more about your life, why you use fentanyl, what that looks like to you, what it feels like to you, what you've tried in the past, what you think will be helpful. You come to me with that knowledge. I don't have that.” *(Staff–substance use physician)*

Appreciation for client’s lived experience as expertise supported prescribers’ transition into safer supply prescribing, and also helped to reduce the stigma associated with substance use. This also led to shifts in what ‘success’ meant to prescribers, moving away from it being defined as abstinence from drugs toward providing safer alternatives and improving quality of life. When asked about what harm reduction meant to them, one physician responded saying:

“My thing was with illicit drug use, was that it was illegal, that it was criminal, that obviously there's a lot of issues around that. But it was also kind of the view of abstinence, like somebody who's a user, I thought they should just stop using. But it's not such an easy process. There's a lot of gray area in it. What I mean is - people shouldn't say that it's bad to use drugs. Yes, yes, it's bad, but it's more like what's circulating in the city that gives it a bad notion. But there should be services that are provided for substance users for them to have a safe space where they're able to use safely, where they have all the information that they need and feel like there would be, that their voices are being heard.” (*Staff–primary care provider*)

### Expanding the definition of evidence-based medicine

The unique challenges of COVID-19 isolation, and the emergency situation in which the CIRS model was developed, may have facilitated prescribers’ willingness to utilize a novel intervention like safer supply that did not yet meet a sufficient standard of evidence to many providers. According to the prescribers we interviewed, the discussion about evidence (and lack thereof) for safer supply had been ongoing among addictions medicine providers, and the pandemic provided impetus to move forward with larger scale prescribing in settings like isolation sites:

“The discussion around safe supply and - at least from the beginning - the chicken and the egg of ‘Well, we can't do the same because we don't have evidence as per our metric of what evidence is’. But if you don't do this, how do you get evidence? So I've seen this sort of circular argument go around a lot. I think COVID really made the onus, more important, that we try something and gather evidence for or against, versus relying on only the things that we had already evidence of. When we're talking about evidence, we're talking about randomized controlled trials and things like that. And I think the truth is there's a push toward innovating a little bit more, and then also relying on evidence from people who use drugs. And acknowledging that there's many different things of what we're calling evidence here.” *(Staff–substance use physician)*

Another prescriber described how the context of two concurrent public health emergencies–the COVID-19 pandemic amidst a continuing and worsening overdose crisis–created conditions in which practitioners had to do their best with the information and evidence available to guide their decision-making around prescribing safer supply:

“There was not clear evidence. And the way I view it as a practicing physician, my patterns and what I decide, I take some evidence in mind, but also I recognize that we've never had these two pandemics at the same time. And so we have to do our best with the knowledge, with the information that we have at hand.” (*Staff–primary care provider*)

Prescribers also highlighted that it was frequent in the practice of medicine to carefully try out novel treatments and approaches, out of a desire to generate evidence through action: “… just because there's no evidence for something doesn't mean you can't try it. And that's because you’re never going to gather the evidence unless we actually do it, right?” *(Staff–substance use physician)* However, many substance use specialists seemed to wrestle with what constituted evidence of success of when prescribing safer supply, particularly in moving beyond abstinence as the gold standard outcome measure for the effectiveness of an intervention:

“I think that the conflict is, I have seen patients who have not changed among the people who I have followed. There have been a couple who have stopped using, but the majority people have not. So I don't know if that's the goal of safer opiate supply. And then I'm not convinced as yet because I haven't seen it be super effective for a lot of people. But it doesn't mean that it's not making their lives a little bit better. Maybe they're using less fentanyl. Maybe they're trading their tablets instead of having to participate in crime to get money to support their fentanyl use. So maybe it is reducing harms in other ways. It's just not obvious.” *(Staff–substance use physician)*

Some healthcare providers–like the substance use specialist above–seemed to have difficulty conceptualizing a harm reduction-oriented expansion in acceptable client goals beyond the predominant focus on abstinence within addiction medicine, to goals like using less fentanyl from the street supply. Additionally, this staff member raised concerns that people may be engaging in diversion of their medication. This frequently repeated yet anecdotal concern may be rooted in stigma based on its assumption that people who use drugs are more likely than other patients receiving opioids to not use medications as intended.

Part of the reticence to move beyond a narrow range of abstinence-based goals seemed to be driven by a hesitation to fully engage with patients who use drugs as people with expertise that could be accounted for in clinical decision making: “There's a whole body of what I would call ‘evidence of human experience’ and ‘experience using drugs’ and all these things. But that's so delegitimized in this model, it’s a very small part of this small particular thing that we think is the only legitimate evidence out there”*(Staff–substance use physician).* There seemed to be considerable variation among both the primary care providers and substance use specialists on how to engage with clients in a way that took into account their experience and expertise with substance use, and that might support a reconceptualization around the goals of care:

“Most things in medicine are kind of like, “I know more about this than you”, and in addictions that's really shifted. It's really actually challenging for us, and I think the concept of things like safe supply and things that are more led by people who have expertise in drug use from themselves is hard for us to sort of reframe the way that we do things, and get out of it.” *(Staff–substance use physician)*

Many prescribers experienced a perspective shift in what constitutes sufficient evidence for clinical-decision making, particularly in the context of concurrent public health crises, prompted by the urgency presented by the COVID-19 pandemic and overdose crisis and facilitated by the collaborative client-centered approach at the CIRS.

## Discussion

This study contributes to the existing research exploring the feasibility and value of low-barrier prescribed safer supply within temporary CIRS for people experiencing homelessness [8, 9]. The unique circumstances presented by the COVID-19 pandemic created an urgent need to facilitate isolation for people who use drugs, and was a major force in accelerating the provision of prescribed safer supply in the Canadian context [12]. In the case of the Toronto CIRS, the interdisciplinary nature of the staff team and the prioritization of harm reduction approaches created an environment that supported the adoption of prescribed safer supply as a critical component of the care provided to people who use drugs in need of COVID-19 isolation. This was made possible by an environment where concrete strategies (including the development of a guidance document and presence of a substance use specialist team) and a philosophy that valued the expertise of clients and supported clinicians to rapidly learn new clinical skills and overcome hesitations around prescribing safer supply. Overall, the clients and staff who were interviewed expressed having positive experiences with safer supply, particularly as a method to facilitate successful completion of COVID-19 isolation periods. Additionally, safer supply was seen as a way to simultaneously reduce the risk of fatal overdoses and substance-use-related issues during isolation. These results align with those found in other CIRS models in Canada [8, 9], while providing additional insight into the elements at play within clinical teams as prescribed safer supply was broadly implemented at the CIRS.

Our results show the value of a harm reduction approach in challenging traditional medical models in which clinicians are the sole experts and abstinence is the sole goal of substance use services. Providers’ comments reflected the importance of understanding the social context of people’s lives and their motivations for drug use to provide successful client-centered care. Relationships between healthcare providers and clients redefined clinical success to be about quality of life and dignity, rather than abstinence from substances. The urgent need to facilitate COVID-19-related isolation allowed clinical providers to embrace an expanded range of acceptable goals for substance use care and influenced willingness among prescribers to acknowledge people’s desire to continue using substances during their isolation. Our findings highlight how the urgency involved in intervening during crisis periods such as the intersection of the overdose crisis and the COVID-19 pandemic can enable novel approaches to meet the needs of people who use substances, and expand acceptable goals in the care of people who use drugs, such as moving beyond requirements for abstinence. Notably, safer supply programs have primarily been implemented in Canada through a medicalized model which requires a doctor or nurse practitioner to prescribe pharmaceutical options based on provincial drug formularies, and which have been criticized as a limited measure compared to broader drug policy changes such as decriminalization or non-prescriber based regulatory models for safer supply [26]. The unique circumstances of the CIRS pushed prescribers beyond their usual practices and provided an impetus to shift from strictly treatment-focused approaches toward more harm reduction-oriented measures like safer supply, that provide access to regulated medications solely as a substitute for the non-regulated, illegal drug supply. This shift provides insight into care delivery and points to the potential of non-medicalized, community-driven models of safer supply to further challenge the structural constraints that shape drug use and meet the needs of people who use drugs. Expanding prescriber views on caring for people who use drugs may have broader implications for opioid use disorder treatment, which has typically been delivered in a treatment model that frames drug use and addiction as an individual-level medical condition (i.e., model of addiction as ‘chronic, relapsing brain disease’) [31]. The framing of drug use and addiction in this way effectively obscures the structural forces that shape drug use and actively cause harm, namely prohibition and criminalization of drugs and people who use them, as well as obscuring how people frequently engage in addiction treatment and seek to obtain opioid agonist treatments such as methadone not because they believe they have a medical disorder, but to access a regulated supply of opioids [32].

Similarly, the CIRS Substance Use Service model may be generalizable to existing hospital and shelter settings to support the care of people who use drugs. Many hospitals do not have inpatient substance use services; additionally, the current range of acceptable interventions for people who use drugs in hospital settings are still very limited, with the provision of short-acting opioids to address pain and withdrawal still considered contentious [33–35]. Many inpatient substance use services may not be equipped to recognize or respond to the diverse underlying motivations for drug use, particularly for patients who are hospitalized and have no desire for cessation of substance use. Our results suggest that clinician acceptance of a wider array of patient goals related to engagement in care and definitions of success may improve the accessibility and acceptability of care, and that offering safer supply is a feasible strategy for supporting patients requiring in-patient treatment to remain in hospital to receive necessary care. Our findings also demonstrate the value of sub-acute medical settings where individuals can achieve greater stability while accessing broader wrap-around health care and social supports. Implementation of sub-acute medical settings with embedded harm reduction practices and values may increase accessibility to health care for people experiencing homelessness whose care needs are too complex to be treated in a shelter setting. For effective implementation, clinical guidance and substance use specialist consults may be helpful to assist healthcare providers, especially those who are new or hesitant about prescribing safe supply. Interdisciplinary teams providing integrated services should include harm reduction workers and peer support. Care provided by all teams should be rooted in a client-centered paradigm that values the experience and expertise of clients and embraces a wide range of care goals that are defined by and with clients.

Prescribed safe supply programs require willing prescribers. In the case of the CIRS, the development of context-specific prescribing guidelines and the on-call substance use specialist consult team were essential to building prescriber capacity and confidence. Our results also highlight the importance of fostering a community of practice in which prescribers with varying levels of experience can engage with and learn from each other. The CIRS was a unique environment because clinicians prescribing safer supply were witnessing and responding to overdoses, which was a factor that accelerated prescriber willingness; healthcare providers frequently expressed increased motivation to prescribe safe supply to protect clients from relying on street drugs which would increase risk of fatal overdoses. In contrast to other addiction medicine settings were overdose response was infrequent or non-existent, healthcare providers at the CIRS directly experienced the link between consumption of the unregulated drug supply and resulting overdoses; this put the harms of not prescribing safer supply into perspective and seems to have facilitated their willingness to prescribe safer supply as an alternative. Our results suggest that the frontline experience of responding to frequent overdose and working closely with harm reduction and peer workers was crucial in altering perspectives and motivating changes in practice. The combination of available guidelines, a specialist consult service, an integrated harm reduction and peer team, and being faced with the immediate impact of safer supply for people at high risk of overdose facilitated a rapid learning environment for new prescribers. These factors may be helpful to consider in other environments seeking to scale-up prescribed safe supply programs. While much opposition to safer supply prescribing centers on the risks of prescribing, ensuring healthcare providers have frontline experience in community settings may provoke new understandings the risks of *not* prescribing safer supply.

Lack of support from regulatory colleges and clinical guidelines are a commonly cited barrier to clinicians prescribing safe supply [27, 36]. Concerns about jeopardizing professional licensure among regulated professionals, being reprimanded by their respective regulatory bodies and being subject to reprobation from mentors and colleagues were all raised by participants as issues that they feared or had actually experienced. However, perspectives on the concerns around implementation of a clinical prescribing practice that is novel and evidence-informed, rather than firmly evidence-based began to shift, due in part to the challenges presented by the COVID-19 pandemic. A guidance document on safer supply prescribing had been released by Ontario clinicians prior to the pandemic [37]; the addition of new guidelines on “Risk Mitigation Prescribing” in the province of BC in April 2020 provided guidance for safe supply prescribing as a mechanism to reduce COVID-19 transmission [29]. Policy changes have continued since, with the province of BC announcing new policy around “Prescribed Safer Supply” in 2021 [38] and the College of Physicians and Surgeons of Ontario releasing a statement which acknowledged safer supply prescribing as an emerging area of clinical practice [39], and funding was announced by the federal government to fund short-term pilot safe supply programs across the country [40]. These developments are indicative of a changing political landscape surrounding safer supply policy and practice in the wake of dual public health emergencies. However, prescribers who are both motivated and willing to prescribe safer supply remain limited in a system with constrained resources and ongoing contention around the practice, despite these recent shifts. Our study adds to the rapidly expanding evidence base demonstrating the positive outcomes associated with safer supply programs, as seen in client health, well-being and stability [15, 16, 20–22, 41], as well as positive impacts on clinical outcomes and health system costs [14]. Given the continuing and unacceptably high rates of drug toxicity overdose deaths in Canada, translating evidence into rapid adoption of safer supply by more prescribers is critical; the approaches outlined in our study may be leveraged for building prescriber confidence and capacity.

A limitation of our study is that a convenience sample of clients was recruited for interviews. The need for participants to be both available and willing to be interviewed may have introduced a source of recruitment bias. Second, results may not be generalizable to other contexts given the narrow scope of the study, including results from one CIRS in Toronto, Canada. Third, interviews occurred during the Delta wave of COVID-19 when Ontario still had significant public health measures in place, a situation which has changed in subsequent COVID-19 waves, and which may have shaped both the intervention and responses. Finally, this study took place at the intersection of rising deaths due to the toxic drug supply, the COVID-19 pandemic and a housing crisis. Though all three of these crises are ongoing, the majority of public health restrictions for COVID-19 are no longer in place. Therefore, some study findings may have been unique to this setting and point in time.

## Conclusion

The rapid implementation of comprehensive substance use services that included prescribed safer supply of opioids and stimulants at the Toronto CIRS emerged as a response to the urgent public health needs created by the COVID-19 pandemic within the context of an overdose crisis. Our findings provide insights into the necessary components for quickly implementing and operating safer supply programs, including the need to provide comprehensive harm reduction services through interdisciplinary teams trained in harm reduction and overdose prevention, and supporting prescribers with context-appropriate clinical guidance and a specialist substance use consult service. The provision of safer supply in this context was feasible and acceptable to both clients and staff. Given the ongoing overdose crisis in Canada, similar services should be expanded to improve health care accessibility and positive health outcomes for people who use drugs. These findings may be applicable to providing safer supply in other contexts, in-patient hospital settings in particular.

## Data Availability

The datasets generated and analyzed during the current study are not publicly available given the sensitive nature of the research topic, as they contain confidential information that could compromise participant confidentiality and consent.
